# Everyday non-partisan fake news: Sharing behavior, platform specificity, and detection

**DOI:** 10.3389/fpsyg.2023.1118407

**Published:** 2023-05-15

**Authors:** Mark P. Shephard, David J. Robertson, Narisong Huhe, Anthony Anderson

**Affiliations:** ^1^School of Government and Public Policy, University of Strathclyde, Glasgow, United Kingdom; ^2^School of Psychological Sciences and Health, University of Strathclyde, Glasgow, United Kingdom

**Keywords:** fake news, social media, news sharing, news sharing platform, individual differences

## Abstract

Concern over the impact of fake news on major socio-political events is growing. The use of deliberate misinformation is thought to have played a role in the outcome of the UK EU referendum, the 2016 US presidential election, and in the effectiveness of COVID-19 public health messaging. As a result, recent research has tended to focus on hyper-partisan (e.g., US politics; Democrat/Republican), person specific (e.g., Hillary Clinton/Donald Trump) content that incorporates emotive and hyperbolic language. However, in this study, we focus on an alternative form of fake news, across a variety of topics (e.g., Crime, Immigration, and Health), that avoids these characteristics, and which may therefore be more pervasive and difficult to detect. In a three-part study, we examined participants sharing intentions for fake news (including platform preference; Facebook, Twitter, Instagram, and WhatsApp), their ability to explicitly detect fake news, and whether individual differences on psychological measures of critical thinking ability, rational thinking, and emotional stability predict sharing behavior and detection ability. The results show that even our well-informed sample (political science students) were not immune to the effects of fake news, some issues (e.g., health and crime) were more likely to be shared than others (e.g., immigration), and on specific platforms (e.g., Twitter, Facebook). In addition, we show that individual differences in emotional stability appears to be a key factor in sharing behavior, while rational thinking aptitude was key to fake news detection. Taken together, this study provides novel data that can be used to support targeted fake news interventions, suggesting possible news topic, sharing behavior, and platform specific insights. Such interventions, and implications for government policy, education, and social media companies are discussed.

## 1. Introduction

The dissemination of misinformation in the form of unsubstantiated rumor and intentionally deceitful propaganda is, and has always been, a ubiquitous feature of society (Posetti and Matthews, 2018). However, the pervasive and damaging effects that misinformation campaigns can have, has been elevated beyond measure by the emergence of social media as a global information platform which encompasses all aspects of daily life (e.g., health, education, and politics). Research on the role of fake news in several recent socio-political events such as the 2016 UK EU Referendum (see Hänska and Bauchowitz, 2017; Bastos and Mercea, 2019; Greene et al., 2021), the 2016 US Presidential Election (see Allcott and Gentzkow, 2017; Guess et al., 2018), and the 2020 COVID-19 pandemic (see Brennen et al., 2020; Kouzy et al., 2020; Greene and Murphy, 2021 Melki et al., 2021) have highlighted the negative impact that it can have. For example, misinformation leads to the loss of public trust in the political class, governing institutions, and democracy (Marshall and Drieschova, 2018; Reglitz, 2021); greater political and public polarization across a range of issues (Bago et al., 2020; Au et al., 2021; Osmundsen et al., 2021), and, in relation to the recent pandemic, poorer public health choices (Naeem et al., 2021; Rocha et al., 2021). Improving the detection of fake news and ameliorating its impact must therefore be a priority area for applied research.

One of the primary forms of fake news is ‘political clickbait’ (Waldrop, 2017). These fabricated, often sensationalized headlines, about political figures or issues, encourage users to click on the post which takes them to the main ‘article’ site. There are two underlying reasons for the creation of such content. The first is political, with individuals or campaigns seeking to attack their opponent, while also drawing users to further digital content which might be advantageous to their own aims (Allcott and Gentzkow, 2017). The second is money, where content creators can enrich themselves through the inclusion of advertising on their fake content sites, with revenue rising in line with site traffic (Braun and Eklund, 2019). In addition, fake news providers rely on users to share and re-post their content for consumption by the 3.8 billion strong social media audience, a task which is also accomplished effectively by the use of automated ‘bots’ (Shao et al., 2017; Bastos and Mercea, 2019), with such repeated exposure to the content increasing its face validity (Hasher et al., 1977; Fazio et al., 2015; Pennycook et al., 2018, 2020; see also Marchi, 2012).

At first glance, one promising route to help users detect fake news would be to use sophisticated machine learning algorithms to detect such content and to add a ‘fake news’ or ‘fact check’ warning to the post (see Rudgard, 2019; BBC News, 2020). However, research has shown that such warnings produce only modest reductions in fake news veracity judgements, and in some cases, they can generate a counter-productive ‘implied’ truth effect (i.e., in which the perceived veracity of untagged fake news items actually increases; Clayton et al., 2020; Pennycook et al., 2020). Therefore, while ‘algorithm-centered’ approaches should continue to be refined, research must continue to focus on ‘user-centered’ approaches to combat the spread and acceptance of fake news content. A user-centered approach focuses on examining the psychological mechanisms that might make one individual more likely than another to click on, accept, and share fake news. In short, the question becomes ‘who falls for fake news and who shares it?’, and if a clear psychological profile can be identified, this provides the opportunity to create interventions which might reduce that likelihood in such individuals (e.g., through educational tools, see Roozenbeek and Van Der Linden, 2019).

Several studies have already utilized a user-centered approach to examine the extent to which human participants can detect fake news. This research typically uses highly political/partisan fake news with deliberately emotive/hyperbolic language (e.g., Corbu et al., 2019; Pennycook and Rand, 2019; Osmundsen et al., 2021), that are often restricted to sets of issues that pertain to specific leaders and/or parties (e.g., Allcott and Gentzkow, 2017). For example, ‘Hillary Clinton’s use of a private e-mail server broke the law, and she should be jailed’. User-centered research has shown that some individuals are better than others at seeing through this type of provocative fake news (see Preston et al., 2021), and this opens up the possibility to train others to do the same. However, many forms of fake news avoid overtly partisan content/provocative language (e.g., ‘COVID lockdown used to trial roll back of civil liberties’) and may therefore be more pervasive and difficult to detect. It is this type of fake news that we focus on in this paper. We posit that such fake news may be more difficult to detect, as it does not have salient partisan markers which might at least cause those of a differing political persuasion to check its veracity. In addition, it may be more pervasive as it is not likely to be tied to specific socio-political events, rather it is likely to relate to everyday topics such as crime, health, and education, for example.

Therefore, in this paper we take a user-centered approach to examine sharing intentions and detection ability for ‘everyday’ non-partisan fake news. We broaden the scope of fake news research by examining a variety of news topics beyond the typical political news set (e.g., US politics; Democrat/Republican) using items from the following topics: Crime, Economy, Education, Europe, Scotland, Health, and Immigration. In a three-part study we asked participants, without any forewarning that the news item set contained fake content, whether they would share the item, and if so, on which platform (Facebook, Twitter, Instagram, and WhatsApp). Following this, we made participants aware that some of the content was fake, and they were then asked explicitly to detect it. In addition, research has shown that some individual differences in psychological metrics might be predictive of fake news detection ability (e.g., see Pennycook and Rand, 2020 for critical thinking aptitude; see Preston et al., 2021 for emotional intelligence), with less focus to date on sharing behavior, and so here we also test participants on tasks which measure critical reasoning ability, rational thinking style, and emotional stability.

Taken together, this approach allows us to test sharing behavior for fake news across a wider range of topics than is usually the case. Any differences that arise between news topics may be informative to researchers seeking to explore and understand wider news sharing behavior for major issues of concern to the public, and importantly, beyond those framed by US presidential politics (e.g., Altay et al., 2022). In addition, *where* users prefer to share news is an understudied area of misinformation research, and so here we asked users, who selected yes to ‘I would share this item’ in the sharing task, what social media platform they would use to disseminate that information. If differences arise across the platforms, this will be particularly useful for researchers and social media companies who are seeking to create targeted interventions for specific platforms and for specific fake news topics (see Guess et al., 2019; Hopp, 2021; Osmundsen et al., 2021). Finally, a key aspect of the user-centered approach to combatting the rise of fake news is to establish who is likely to share it and who is better at detecting it. To that end, we use the critical reasoning task (CRT; Frederick, 2005; see Pennycook and Rand, 2020), the Norris and Epstein Rational Thinking Scale (Pacini and Epstein, 1999) and the emotional stability sub-test of the IPIP Big-5 personality scale (see Gow et al., 2005) to examine whether individual differences on these measures predict sharing behavior and news veracity judgements. The latter two tests, to our knowledge, have not previously been used in fake news research, and should an effect arise, this will further enhance researchers understanding of the type of psychological profile that leads some individuals to have a greater propensity to fall for fake news than others. Such profiles in turn, could support targeted user interventions (see Roozenbeek and Van Der Linden, 2019; Preston et al., 2021).

## 2. Materials and methods

### 2.1. Ethics statement

This study received concurrent approval from the ethics committees of the School of Psychological Sciences and Health, and the School of Government and Public Policy.

### 2.2. Participants

A G*Power analysis (Faul et al., 2009), based on a multiple linear regression approach, with alpha set at 0.05, power set at 0.80, and *f*^2^ at 0.15, indicated that a minimum of 77 participants would be required to detect an effect of that size. Therefore, we recruited 100 undergraduate student participants from the School of Government and Public Policy, 18 participants were excluded based on a failure to provide informed consent (*N* = 4) or failing to complete, at minimum, the first two sections of the study (*N* = 14), providing a final sample of 82 participants with a mean age of 20 years (SD = 4, *Range* = 17–44; 54% Female). Facebook accounted for most of the participants social networking accounts (95%), followed by Instagram (87%), Twitter (73%), and WhatsApp (48%). Self-reported usage of these platforms included study relevant terms such as ‘accessing news’ (67%), ‘keeping up with current affairs’ (57%), ‘engaging in political debate’ (15%). Participants reported talking to other people about politics and the news on 4 days out of the last seven, on average, with the majority of participants (83%) spending up to a minimum of 1 hour per day specifically seeking political news and information online. Most of the participants identified with the main left of center UK political parties (SNP 35%, Labour 31%, Conservatives 10%, Liberal Democrats 7%, Greens 7%, UKIP 3%, Other 7%). As expected, our sample of political science undergraduate students show a high level of engagement with online information/news seeking and political discourse and should therefore be well placed to detect non-partisan fake news (relative to the general population). All participants received £15 on completion of the study to reimburse them for their time.

### 2.3. Measures

#### 2.3.1. Sharing behavior task

This task consisted of 21 real news items and 21 fake news items that were sourced from fullfact.org, the Scottish Government website, and generated by the authors. Note that this item set was created and used for testing in 2019–2020 and reflects relevant news topics at that time. There were seven different news topics: Crime, Economy, Education, Europe, Scotland, Health, and Immigration. For each topic there were three real and three fake news items (see Supplementary materials for full list). Care was taken to ensure, as far as possible, that the items avoided hyperbolic and overt partisan language and content. In line with fake news content creators, plausible fake news items were created by focusing on real issues and altering the direction or exaggerating the magnitude of the core message (e.g., inflating numerical figures/costs for real issues, for example, with Education, the educational maintenance payment amount). Participants were asked to read each item, presented in simple text format, carefully and to report whether they would share the news item, and if so, which social media platform they would use (the response options were: Facebook; Twitter; Instagram; WhatsApp; Would not share).

#### 2.3.2. Psychometrics

##### 2.3.2.1. The cognitive reflection test

The CRT is designed to measure the tendency to override an initial intuitive incorrect response and to engage in further critical analysis and reflection that leads to the correct answer (Toplak et al., 2012). In this study we used the three-item version of this test, and example item is: ‘A bat and a ball cost $1.10 in total. The bat costs $1.00 more than the ball. How much does the ball cost? _____ cents.’ See Frederick (2005) for full details.

##### 2.3.2.2. Rational Thinking Style

This task consisted of 10 questions that assessed whether an individual was more likely to adopt a rational thinking style. Each item was paired with a 7-point Likert scale ranging from ‘1’ Not at all to ‘7’ Very much so. An example item is: ‘I enjoy problems that require hard thinking’. For full details of this test (see Pacini and Epstein, 1999).

##### 2.3.2.3. Emotional stability

We used the 10-item emotional stability scale from the IPIP Big 5 personality measure (see Gow et al., 2005). Participants responded to each item using a 7-point Likert scale ranging from ‘1’ Not at all to ‘7’ Very much so. An example item is: ‘I get upset easily (this item is reversed scored)’.

#### 2.3.3. News veracity task

In this task, participants were made aware that some of the items they encountered during the sharing task were fake news. The participants were then presented with each of the 42 news items once again, and this time they were asked to report, using a seven-point Likert scale (1 Real News, 7 Fake News), how likely it was that the item was fake.

### 2.4. Procedure

Qualtrics, the online testing and data collection platform, was used to present the study to participants and to collect the data. All participants completed each part of the study in a fixed order: Sharing Behavior Task, Psychometrics, and Fake News Detection Task. The order in which each item within a task appeared was randomized. The study was self-paced, but participants were asked to complete the study within 1 h, with time factored in for screen breaks.

## 3. Results

### 3.1. Sharing behavior: Content and veracity

We start by assessing real and fake news sharing rates for the different news topics aggregated across the social networking platforms. To that end, participants mean percentage sharing rates were entered into a 7 × 2 repeated measures Analysis of Variance (ANOVA) with the factors of news topic (Economy, Europe, Immigration, Scotland, Crime, Health, Education) and news veracity (Real, Fake).

The ANOVA revealed a main effect of news topic, *F*(6, 486) = 11.72, *p* < 0.001, *η*_p_^2^ = 0.13, and while Health was numerically the most frequently shared topic (*M* = 37%), sharing rates were statistically similar for Crime (*M* = 34%), Education (*M* = 33%), Europe (*M* = 33%) and Scotland (*M* = 31%; all *t’s* ≤ 1.41; all *p’s* ≥ 0.074), followed by news relating to the Economy [*M* = 30%; *t*(81) = 2.76, *p* = 0.007, *d* = 0.31 for the comparison with Health], with participants proving to be most reluctant to share Immigration related content [*M* = 17%; *t*(81) = 4.53, *p* < 0.001, *d* = 0.50 for the comparison with Economy].

**Figure 1:**
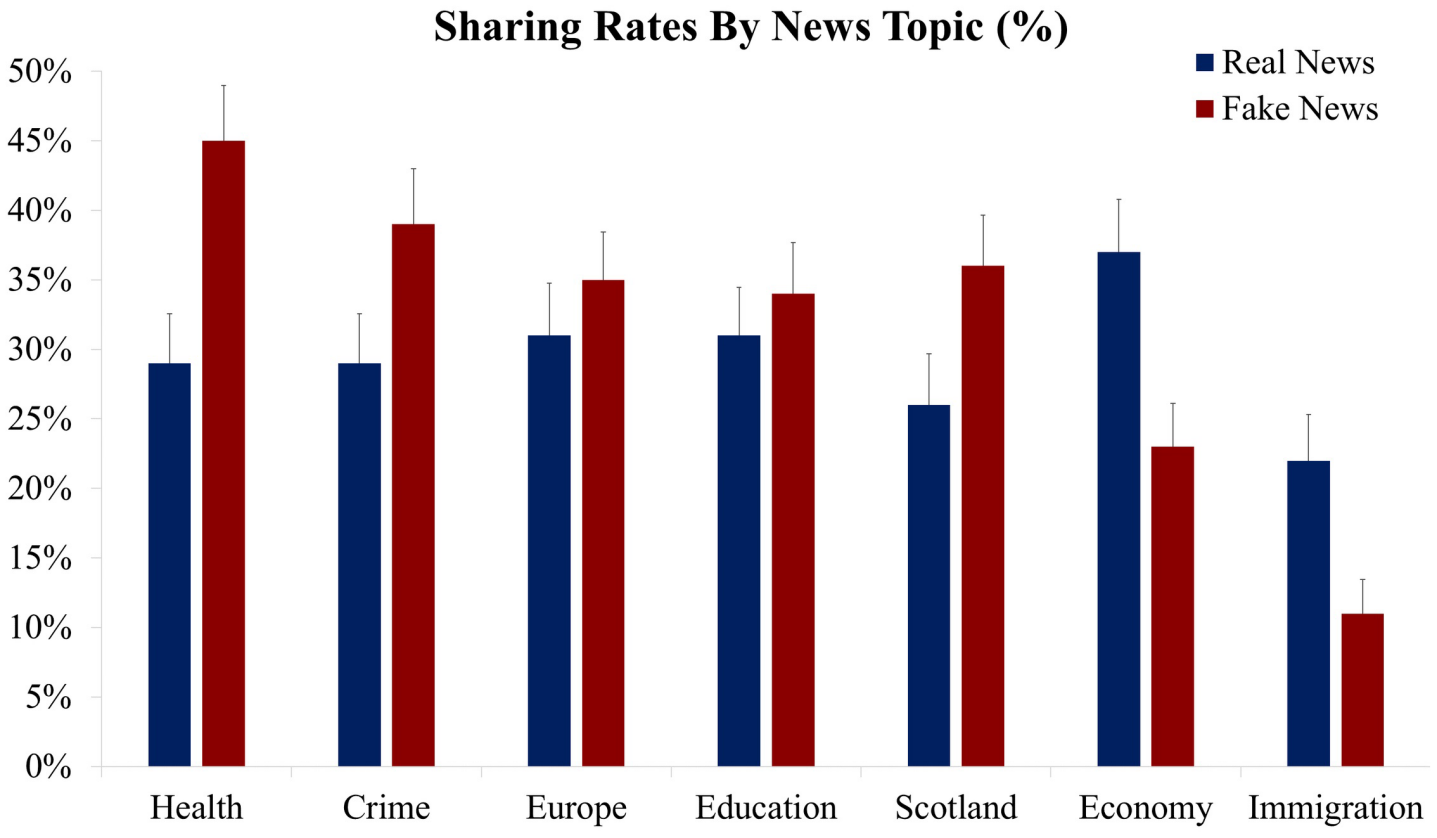
News sharing rates presented as a function of news topic and veracity. Mean percentage news sharing rates (the error bars denote the standard error of the mean).

While there was no overall main effect of news veracity (*M* = 30% for real news; *M* = 32% for fake news), *F*(1, 81) = 2.68, *p* = 0.106, *η*_p_^2^ = 0.03, there was a veracity × topic interaction, *F*(6, 486) = 8.87, *p* < 0.001, *η*_p_^2^ = 0.10. As seen in Figure 1, sharing rates were greater for fake news compared to real news for Health, *t*(81) = 3.95, *p* < 0.001, *d* = 0.44, Crime, *t*(81) = 3.17, *p* = 0.002, *d* = 0.35, and Scotland, *t*(81) = 2.41, *p* = 0.018, *d* = 0.27, with non-significant trends in that direction for Education and Europe, both *t*’s < 1. For the remaining two topics, the direction of sharing behavior was reversed, with significantly greater real news sharing compared to fake news sharing rates for Economy, *t*(81) = 3.79, *p* < 0.001, *d* = 0.42, and Immigration, *t*(81) = 3.52, *p* = 0.001, *d* = 0.39.

These findings show that participants would have shared both real and fake news approximately 30% of the time, on average, and that out of our seven news topics, it appears as though Health related news content was most likely to be shared while the opposite effect was found for content relating to Immigration. This suggests an interesting degree of dissociation in which people are engaging in some form of self-censorship in terms of what topics, or items, they would feel comfortable sharing. For Immigration news in particular, it suggests that misinformation attacks in relation to this topic, may only be successful if it is advertized to, and promoted by, those who already share strong doctrinal feelings about it.

Overall, participants were more often than not likely to share fake news than real news. Interestingly, this effect was largely being driven by a greater propensity to share fake health news compared to real health news. This is concerning, and it confirms that verifying health related news should be an important focus in preventing the spread of health-related misinformation. The higher rates of health-related fake news sharing could be explained by the nature of the user audience. For example, more so than the other news topics, fake health news may be shared both for doctrinal and intentionally misleading reasons (i.e., those knowingly promoting fake health news) *and* by those with the best of intentions (i.e., those seeking to ensure they are sharing important health related information), doubling the prospective sharing capacity.

### 3.2. Sharing behavior: Social networking platforms

**Figure 2:**
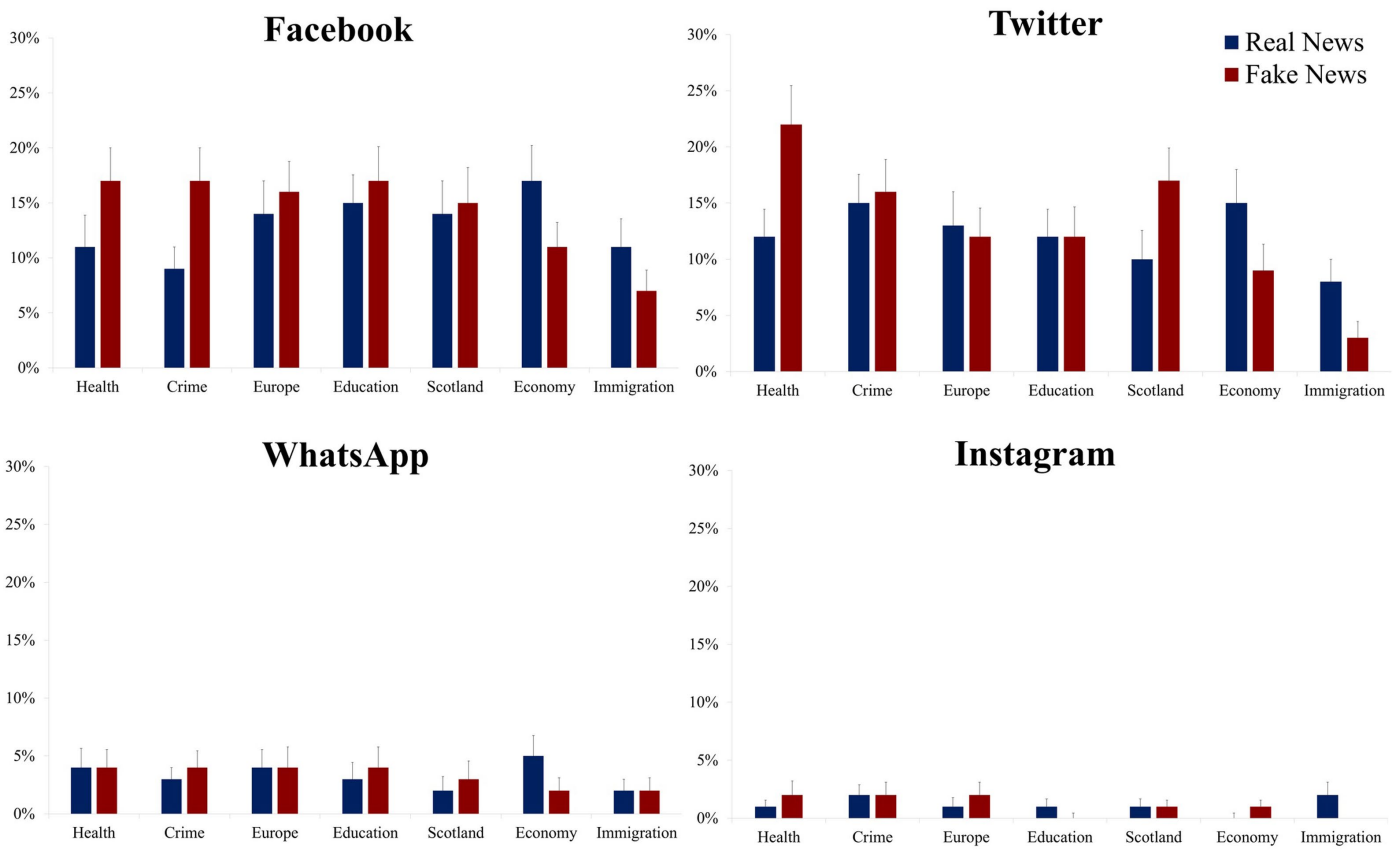
News sharing rates presented as a function of social media platform, news topic, and veracity. Mean percentage news sharing rates (the error bars denote the standard error of the mean).

In this section we examine sharing behavior in relation to veracity and topic within each sharing platform. As seen in Figure 2, participants were most likely to use Facebook (*M* = 14%) and Twitter (*M* = 13%) for news sharing with a very low likelihood of sharing on WhatsApp (*M* = 3%) or Instagram (*M* = 1%).

To maximize the clarity in the reporting and interpretation of the results we use separate two-way ANOVAs rather than using a three-factor analysis. Therefore, for each platform, we examine sharing behavior using a two-way repeated measures ANOVA with the factors of news topic (Economy, Europe, Immigration, Scotland, Crime, Health, Education) and news veracity (Real, Fake).

#### 3.2.1. Facebook

For Facebook, as shown in Figure 2, the ANOVA revealed a main effect of news topic, *F*(6, 486) = 2.61, *p* = 0.017, *η*_p_^2^ = 0.03, with statistically similar sharing rates for Education (*M* = 16%), Europe (*M* = 15%), Scotland (*M* = 15%), Health (*M* = 14%), Economy (*M* = 14%) and Crime (*M* = 13%), with the interesting effect showing significantly lower sharing rates for Immigration (*M* = 9%) in comparison to each of the other topics, all *p’s* ≤ 0.033, with the exception of a marginally non-significant difference with Crime, *t*(81) = 1.91, *p* = 0.060, *d* = 0.21. While there was no overall main effect of news veracity, *F* < 1, there was a veracity × topic interaction, *F*(6, 486) = 3.18, *p* = 0.005, *η*_p_^2^ = 0.04. As seen in Figure 2, the source of the interaction was significantly greater sharing rates for fake news compared to real news for Crime, *t*(81) = 2.83, *p* = 0.006, *d* = 0.31, with a non-significant trend in the same direction for Health, *t*(81) = 1.62, *p* = 0.109, *d* = 0.18, while real news was more likely to be shared than fake news for the Economy, *t*(81) = 2.35, *p* = 0.021, *d* = 0.26, with a non-significant trend in the same direction for Immigration, *t*(81) = 1.94, *p* = 0.056, *d* = 0.21. There were with no significant effects of veracity for any of the remaining news topics (i.e., Europe, Education and Scotland, all *t’s* < 1).

#### 3.2.2. Twitter

For Twitter, as shown in Figure 2, the ANOVA revealed a main effect of news topic, *F*(6, 486) = 6.80, *p* < 0.001, *η*_p_^2^ = 0.07, and while Health was numerically the most frequently shared topic (*M* = 17%), sharing rates were statistically similar for Crime (*M* = 15%), Scotland (*M* = 13%), Europe (*M* = 13%), Education (*M* = 12%), Economy (*M* = 12%), and in line with sharing rates on Facebook, the lowest sharing rates on Twitter were for Immigration related news (*M* = 6%; all *t’s* > 3.44, all *p’s* ≤ 0.001 for each of the related comparisons). While there was no overall main effect of news veracity, *F*(1, 81) = 1.41, *p* = 0.239, *η*_p_^2^ = 0.02, there was a veracity × topic interaction, *F*(6, 486) = 5.35, *p* < 0.001, *η*_p_^2^ = 0.06. As seen in Figure 2, the source of the interaction was significantly greater sharing rates for fake news compared to real news for Health, *t*(81) = 3.71, *p* < 0.001, *d* = 0.41, and Scotland, *t*(81) = 2.63, *p* = 0.010, *d* = 0.29, while the opposite pattern was found for Economy, *t*(81) = 2.11, *p* = 0.038, *d* = 0.23, and Immigration, *t*(81) = 3.14, *p* = 0.002, *d* = 0.35. There were no significant effects of veracity for any of the remaining news topics (i.e., Crime, Europe and Education, all *t’s* < 1).

#### 3.2.3. WhatsApp and Instagram

For Instagram, as seen in Figure 2, the ANOVA revealed no main effect of news topic, *F*(6, 486) = 1.07, *p* = 0.378, *η*_p_^2^ = 0.01, veracity, *F* < 1, and there was no topic × veracity interaction, *F*(6, 486) = 1.43, *p* = 0.201, *η*_p_^2^ = 0.02, and this pattern of effects was the same for WhatsApp, for topic, *F*(6, 486) = 1.63, *p* = 0.136, *η*_p_^2^ = 0.02, veracity and interaction, both *F*’s < 1.

Taken together, the platform analysis findings provide important insights into sharing behavior. It is clear, that while WhatsApp and Instagram play an important role in the growing social networking landscape, there is still a preference for Facebook and Twitter when it comes to topical news information sharing. While a focus on Education and Europe and related news sharing was evident, this could be linked to the priorities of our left-of-center student sample and the study being conducted during heavy coverage of Brexit and what effect a ‘no deal’ with the EU could have on trade and the economy. However, Health related news dominated sharing behavior for Twitter and Facebook, albeit to a lesser extent for the latter, suggesting, in line with the preceding results that this topic, and these platforms, should be an important focus for targeting health misinformation going forward. Conversely, as noted for the previous analysis, immigration related fake news was shared infrequently across the platforms and this could indicate that those seeking to share disinformation about this topic may only find success among those with a doctrinal interest, in other words, the ‘best of intention’ users are not likely to share immigration related content.

### 3.3. Sharing behavior: Individual differences in psychometrics

To maximize statistical power and to reduce the likelihood of Type 1 errors, we collapse sharing rates across the individual topics to create three dependent variables: overall sharing rates (regardless of veracity), overall real news sharing rates (collapsed across topic), and overall fake news sharing rates (collapsed across topic). Here, using a multiple regression analysis, we examine whether individual differences in our psychometric measures (i.e., Critical Thinking, Rational Thinking, and Emotional Stability), presented in Table 1, are predictive of the variance in news sharing rates. These predictor variables did not violate any of the multicollinearity checks, confirming that each contributed enough unique variance to be retained in the analysis.

**Table 1:** Scores on the individual difference measures.

	Psychometric tests			Mean	SD	Range
Cognitive reflection test	43%	38%	0–100%
Rational thinking score	43	11	19–64
Big 5-emotional stability score	37	12	10–68

Each regression model was significant, *F*(3, 71) = 3.93, *p* = 0.012, *R^2^* = 0.142 for overall sharing, *F*(3, 71) = 2.98, *p* = 0.037, *R^2^* = 0.112 for real news sharing, and *F*(3, 71) = 4.49, *p* = 0.006, *R^2^* = 0.160 for fake news sharing, with the predictor variables accounting for around 14%, on average, of the variance in news sharing behavior. However, for each of the models, only the individual differences in emotional stability scores were significantly associated with variance in the sharing rates, and the association was negative (*β* = −0.333, *p* = 0.004 for overall news sharing; *β* = −0.279, *p* = 0.015 for real news sharing; *β* = −0.363, *p* = 0.001 for fake news sharing). In other words, while neither critical thinking ability (CRT), nor a rational thinking style predicted sharing behavior, level of emotional stability did, and it is the case that the less emotionally stable an individual was the more likely they were to engage in news sharing behavior, regardless of its veracity.

### 3.4. Sharing behavior results: Summary

The findings from the sharing behavior data show clear preferences for the type of content which participants indicated they would share, their preferred platforms for doing so, and a link between individual differences in emotional stability and likelihood to share. Next, we turn to participants’ ability to accurately discern real news from fake news.

### 3.5. News veracity task performance

The fake news task used a seven-point Likert response scale with a response of 1 indicating that the participant was sure that the item was ‘real news’ and a response of 7 indicating that the item was highly likely to be ‘fake news’. For real news we categorized any 1–3 response as a correct response to a real news item (5–7 as incorrect), while for fake news we categorized any 5–7 response as a correct response to a fake news item (1–3 as incorrect), and any response to the mid-point value (4) was coded as a ‘do not know’ response, indicating that the participant was not able to made a judgement on the likely veracity of the item one way or the other. The proportion of ‘Do not Know’ responses was relatively low (*M* = 14%), which means that participants felt that most items provided enough information to make a veracity judgement, and there was no overall difference in this type of response across the real and fake news conditions (*M* = 13% for real; *M* = 14% for fake; *F* < 1 for the difference). First, we report participants’ performance in correctly attributing a ‘real’ response to real news content, this is to provide an overview of performance across news topics for this veracity condition, then we focus on our main measure of interest, the extent to which fake news content has been miscategorized as real.

#### 3.5.1. Real news

**Figure 3:**
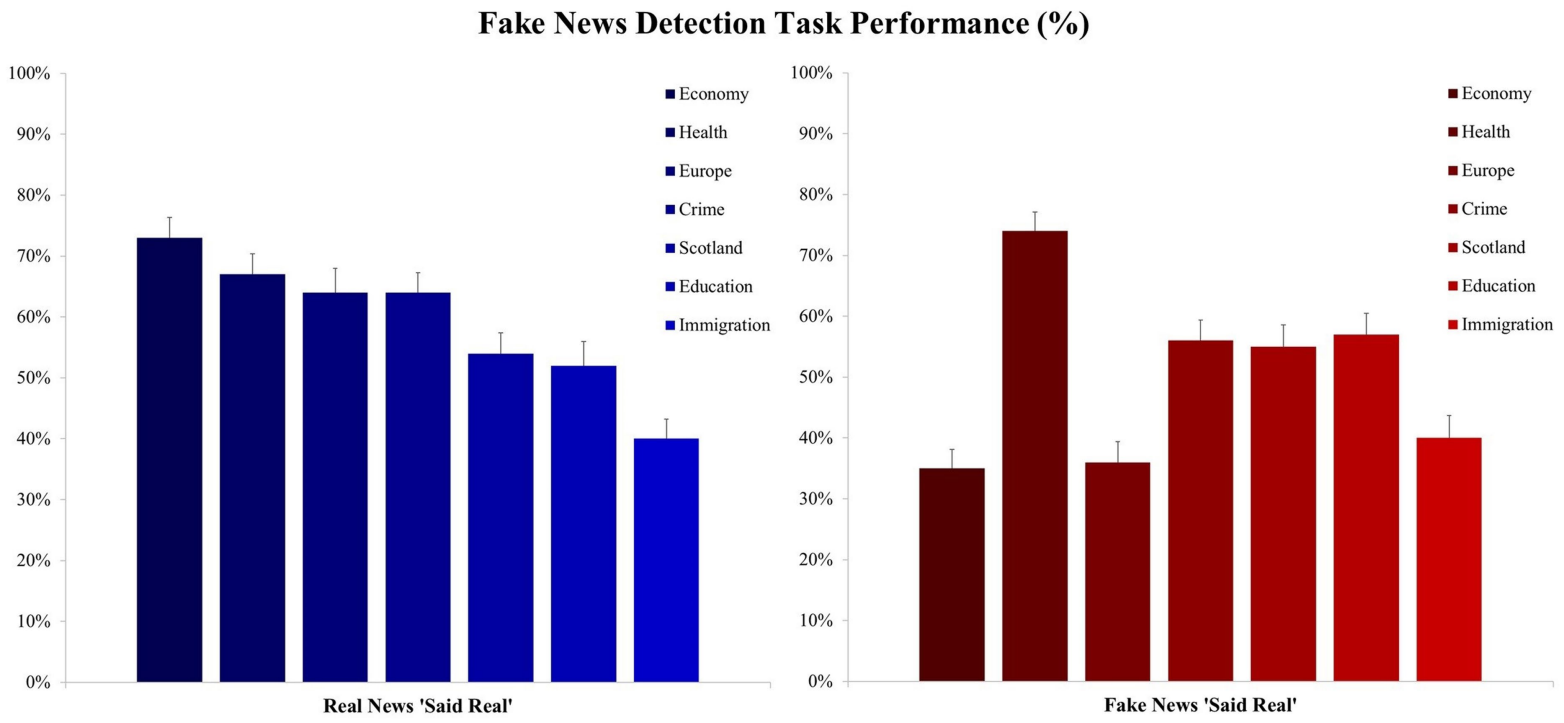
Performance on the news veracity task presented as a function of news topic and veracity. Mean percentage correct veracity attributions (i.e., correctly labelling real news as real and fake news as fake; the error bars denote the standard error of the mean).

For the real news content, participants mean percentage correct responses were entered into a one-way repeated measures ANOVA with the factor of news topic (Economy, Europe, Immigration, Scotland, Crime, Health, and Education). The ANOVA revealed a main effect, *F*(6, 438) = 12.66, *p* < 0.001, *η*_p_^2^ = 0.16, and as seen in Figure 3, real news relating to the Economy (*M* = 73%) was the most accurately labelled, followed by a cluster of Health (*M* = 66%), Europe (*M* = 64%), and Crime (*M* = 64%; all *p’s* ≤ 0.056 for the difference with Economy), followed by Scotland (*M* = 54%) and Education [*M* = 52%; *t*(73) = 2.69, *p* = 0.009, *d* = 0.31 for the difference between Crime and Scotland], with Immigration related news being the least accurately labelled topic for real news items [*M* = 40%; *t*(73) = 2.85, *p* = 0.006, *d* = 0.33 for the difference between Immigration and Education]. These findings, in relation to real news content, show clear differences in participants’ attributions of veracity across news topics. It is somewhat surprising that there were relatively low correct attribution rates for Scotland and Education given the participant sample, however the interesting finding relates to the apparent aversion to attribute veracity to Immigration related real news content, with 60% of the real news items being incorrectly labelled as fake.

#### 3.5.2. Fake news

For the fake news content, we are interested in the extent of the misattribution of veracity across topics (i.e., responding ‘real’ to fake content), and so participants mean percentage incorrect responses to the fake news content were entered into a one-way repeated measures ANOVA with the factor of news topic. The ANOVA revealed a main effect, *F*(6, 438) = 22.15, *p* < 0.001, *η*_p_^2^ = 0.23, and as seen in Figure 3, Health related news generated the greatest proportion of veracity misattributions, with 74%, on average, of the fake Health news content being incorrectly judged as real. This was followed by a cluster of Education (*M* = 57%), Crime (*M* = 55%), and Scotland (*M* = 55%; all *p’s* < 0.001 for the difference with Health), and by a cluster of Immigration (*M* = 40%), Europe (*M* = 35%), and Economy [*M* = 35%; *t*(73) = 4.74, *p* < 0.001, *d* = 0.55 for the difference between Economy and Scotland].

Taken together, the interesting effects from these findings show that participants were most confident in discerning the veracity of news related to the Economy, there is a concerning effect for the misattribution of veracity to fake Health news over and above the other topics, and participants appear reluctant to label Immigration related news as real from either veracity condition. This pattern of findings, for Health and Immigration, also mirror the results from the sharing behavior task.

### 3.6. News veracity: Individual differences in psychometrics

Here we examine whether individual differences in our psychometric tests are associated with news veracity judgements. In line with the sharing behavior analysis, we collapse the data across topic, and here we collapse it across veracity to generate a single dependent variable which reflects participants overall ability on the news veracity task (i.e., the proportion of correct responses to all items in the set). Using CRT, Rational Thinking, and Emotional Stability scores as the predictor variables, the overall regression model was significant, *F*(3, 71) = 3.91, *p* = 0.012, *R^2^* = 0.14, accounting for around 14% of the variance in news veracity aptitude. However, further analysis showed that only the individual differences in rational thinking scores contributed significantly to the model (*β* = 0.277, *p* = 0.015), while there were trends in the data in the same direction for critical thinking (CRT; *β* = 0.156, *p* = 0.166) and emotional stability (*β* = 0.141, *p* = 0.206) these did not reach significance.

This finding suggests that the ability to think rationally may have some predictive value in determining individual differences in the ability to discern real news from fake news. While the CRT has previously been shown to provide an indication of news veracity aptitude, although the effects have tended to be small, here there are trends in our data in that direction. Previous research has shown that emotional traits, in particular the ability to regulate one’s emotions is likely to support greater fake news detection performance, here we do not replicate that finding. However, that is perhaps to be expected, given that our news item set was deliberately chosen to avoid overtly partisan content, this meant a reduction in the emotive and hyperbolic language used, and so, interestingly, emotion related personality traits may not provide a performance advantage for this type of news content (see Preston et al., 2021).

### 3.7. News veracity and sharing behavior

Finally, we explore whether there is any relationship between sharing behavior and individual differences in news veracity performance. To that end, participants overall news sharing rates and overall news veracity accuracy scores were entered into a Pearson’s correlation analysis. The findings showed no significant association in the propensity of participants to share news and their ability to discern real from fake content, *r*(74) = −0.029, *p* = 0.803, this is an important dissociation as it shows individuals who frequently share news can be both good and bad at judging its veracity, and it is the latter group that must be targeted with interventions if we are to reduce the diffusion of misinformation across social networks.

## 4. Discussion

In this study, we focused on an alternative form of fake news, one which might be more widespread, pervasive, and difficult to detect. In doing so, we used a news item set that tried to avoid the highly salient, emotive, hyperbolic, and the overtly partisan fake news (e.g., US Politics) that accompanies major socio-political events (e.g., a US Presidential election), which has been the focus of recent research on misinformation. In addition, where such studies have tended to focus on either fake news detection or, to a lesser extent, sharing behavior, here we included both components, as well as collecting important data on users preferred platform for news sharing, and individual differences in psychological characteristics. Overall, we found, across a range of issue domains (Crime, Economy, Education, Europe, Scotland, Health, and Immigration), that even in our well-informed sample (i.e., university educated political science students), the prospective sharing of fake news items was common enough to be concerning, and the ability to discern fake news from real news was generally poor. Importantly, we also provide some novel data which suggests that emotional stability may be a driver of news sharing behavior, while rational thinking aptitude may be a key predictor in correctly judging fake news from real news.

For the news sharing component of the study, we report that users would have likely shared 32% of the fake news items that they were presented with. This part of the study was intended to emulate the likely experience of users who encounter news on social media, and who make a momentary choice to share an item. We enhanced the ecological validity of this task by making no mention that this was a study relating to fake news at the outset of the experiment, and in doing so, we sought to capture the likely real-world approach/intentions of the users. Previous studies that have focused on partisan news have shown very low sharing rates (e.g., Guess et al., 2019), and reluctant sharing behaviour (e.g., Altay et al., 2022). The reason behind this was thought to be to ensure that the user’s reputation was not damaged among their social networks. However, here we show, that for nearly one third of the fake news content, users decided (in advance of being made aware that some of the content was fake) to share it. This supports our point, that such fake content has the potential to be shared frequently, and it is just as important to create interventions to combat this type of content as it is for those related to the more studied issues of elections or referenda, for example.

For sharing behavior, we also add novel data to the literature by showing that the topic issues of Health and Immigration produced the most interesting effects. The sharing of both real and fake health related content contributed towards a sizable proportion of the shared items, while in contrast, sharing rates, both real and fake, were low for news based on the topic of immigration. In addition to providing applied researchers with data on which topics are likely to be most susceptible to sharing, this finding also generated a potentially interesting dichotomy in users, which has not previously been addressed in the literature. That dichotomy is between users who share fake news because it fits with their world view (i.e., sharing for doctrinal reasons), and ‘best of intentions’ users, those individuals who feel the need to share important content that could be useful to others in their networks. This is manifested in the high sharing rates for real and fake health news, in which both types of users combine to increase sharing rates (i.e., doctrinal; I knew I was right about that issue and ‘best of intentions’; my network need to know about this). In contrast, the low sharing rates for immigration might be explained by that content only appealing to users with a specific doctrinal interest in such news. Going forward, research on fake new sharing behavior, should focus on both types of users to better understand sharing intentions, and our findings suggest that targeting the ‘best of intentions’ group might be an effective way to reduce the unintended sharing of misinformation (see Talwar et al., 2019 for a further perspective on this).

If researchers, companies, and government agencies are going to effectively target and reduce the spread of misinformation, then it is also critical to identify the social media platforms that users show a preference for. In this paper, when a participant indicated, in the sharing task, that they would share a news item, they were asked which social media platform they would use, with the response options being Facebook, Twitter, Instagram, and WhatsApp. In doing so we intended to capture behavior in relation to the most widely used public-facing platforms (Facebook, Twitter, Instagram), as well as a platform that would more readily be used to share information with personal private-facing networks (i.e., WhatsApp). For the public-facing platforms, our findings show that, despite the increasing popularity of Instagram, Facebook and Twitter are the preferred platforms for news sharing. Fake health and crime news was more likely to be shared on Facebook, than real news, and fake health news showed a pronounced sharing effect for Twitter. The latter suggests, particularly following the COVID-19 pandemic, that Twitter should be the focus of fake news interventions in relation to effective health messaging (see Apuke and Omar, 2021). Users indicated that they would share few news items, real or fake, on the private-facing platform (WhatsApp), this might indicate, in line with Altay et al. (2022) a reluctance to attribute contentious content to one’s own reputation among close contacts. We should note, that while a strength of the present study is the inclusion of several of the major social media platforms, we did not include an exhaustive list of all available platforms. Future research, focusing on platform specific interventions, should therefore include a wider range of platforms, particularly Snapchat and TikTok, which are popular with younger users.

In the explicit fake news judgment task, we report considerable variation by topic domain and veracity. For real news detection, users scored above 50%, on average, for each of the topics except immigration. Given the left-of-center bias of our student sample, there may be relatively more reluctance to believe any type of news in relation to immigration, as it is normally only encountered within negative contexts in the media. For fake news detection, a clear effect shows that for fake health content, 74% of users, on average, misattributed these items as being real news. Again, as mentioned above, considering the pandemic, and the importance of effective health messaging, this data supports the notion that combatting health related misinformation is a necessary and pressing problem. Users were better at disregarding fake news related to Europe and the economy, and this is likely to be due to the focus on Brexit and its economic effects which were widely covered in the media during the study. Despite the nature of our sample (undergraduate political science students studying in Scotland), participants were poor at correctly labeling news related to Education, Scotland, and Crime, which suggests, for the first two topics, that closeness to the issues does not always inoculate against the acceptance of misinformation. Overall, in relation to the sample, we would expect the rate of misattribution of fake news as being real, to be greater in the wider population.

As described in the introduction, a further key element of the ‘user-centered’ approach to combating the spread of fake news, is an understanding of the psychological characteristics that make one individual more likely than another to share and fall for fake news. Our findings generate an interesting distinction in that respect. While much of the focus has been on which psychological characteristics predict ‘who falls for fake news’, here we show that individual differences in emotional stability may predict ‘who shares fake news’. Here we report that the less stable a user is in regulating their emotions, the more likely they are to share fake news. More research is needed on this, however, based on this finding, a simple intervention could be developed which tests individuals on this metric, alerting low scorers to be mindful of their news sharing behavior. This finding is also consistent with recent work by Preston et al. (2021) who showed that lower emotional intelligence reduced fake news detection rates, and so future research should focus further on how emotion, and perhaps more broadly, mood states, affect sharing intentions. In contrast to the findings from the sharing task, rational thinking ability was the key predictor of the accuracy of news veracity judgments. This finding builds on previous work (see Pennycook and Rand, 2020), and suggests that the assessments of rational thinking ability should be included in studies which examine how individual differences in psychological characteristics affect the ability to judge the veracity of fake news. In addition, developing ways to enhance rational thinking, particularly in young people who are encountering news based social media for the first time, could also provide a user-centered means for reducing the efficacy of fake news content (see Toplak et al., 2012). However, as we noted in the Introduction, that user-based interventions should also work in tandem with improvements in fake news detection algorithms, and machine-based solutions that work to prevent the dissemination of fake news before it reaches the user’s newsfeed.

To conclude, in this paper we sought to examine an understudied, but important, facet of the wider fake news and misinformation debate. Namely, the type of everyday non-partisan fake news, across a variety of topic issues, that content creators may generate to further their own narratives. We argue that such news is as deserving of focus as that which surrounds the major socio-political events, and in particular, our findings show that combatting health related misinformation must be a priority area going forward. We show that sharing rates for such news, even among a well-informed sample, is concerningly high, that correctly attributing fake news as fake was far from robust across the topics, that there are important dissociations that need to be considered in relation to user intentions (i.e., doctrinal vs. ‘best of intentions’), and the psychological drivers of sharing (i.e., emotion) and detection behavior (i.e., rational thinking). Taken together, the findings from this paper should provide those seeking to develop fake news interventions with new data relating to this type of everyday fake news, sharing platform preference, and individual differences, that might reduce the sharing of misinformation and increase the detection of fake news among most social media users, who are not seeking to propagate, or fall for, fake news.

## Data availability statement

The data that support the analysis reported in this paper is available from the corresponding author upon reasonable request.

## Ethics statement

The studies involving human participants were reviewed and approved by University of Strathclyde School of Psychological Sciences and Health Ethics Committee and School of Government and Public Policy Ethics Committee. Written informed consent to participate in this study was provided by the participants.

## Author contributions

All authors listed have made a substantial, direct, and intellectual contribution to the work and approved it for publication.

## Funding

The research reported in this paper was supported by a WhatsApp Research Award for Social Science and Misinformation to authors NH and MS. The funders had no role in any aspect of the experimental design, data collection, analysis, or manuscript preparation.

## Conflict of interest

The authors declare that the research was conducted in the absence of any commercial or financial relationships that could be construed as a potential conflict of interest.

## Publisher’s note

All claims expressed in this article are solely those of the authors and do not necessarily represent those of their affiliated organizations, or those of the publisher, the editors and the reviewers. Any product that may be evaluated in this article, or claim that may be made by its manufacturer, is not guaranteed or endorsed by the publisher.
