# Supplementary material for: Everyday non-partisan fake news: Sharing behavior, platform specificity, and detection
Source: Front Psychol. 2023 May 15;14:1118407. doi: 10.3389/fpsyg.2023.1118407 (PMC10225687; doi:10.3389/fpsyg.2023.1118407)
Supplement: Supplementary file 1 [file Table_1.DOCX]

Supplementary Material

**Everyday non-partisan fake news: Sharing behavior, platform specificity, and detection**

**David J. Robertson^1^, Narisong Huhe^2^, Anthony Anderson^1^,** **Mark P. Shephard^2^***

***Correspondence:** Mark P. Shephard ([mark.shephard@strath.ac.uk](mailto:mark.shephard@strath.ac.uk))

# Supplementary Data

| **Real News** | **Fake News** |
| --- | --- |
| Economy | Economy |
| Rail commuter fares are rising faster than wages | UK pensions are small because our National Insurance payments are being used to reduce the national debt |
| Youth unemployment is down 44% since 2010 | The UK’s deficit is reduced by Scottish contributions to the UK GDP |
| The working week in France is 35 hours per week | Since 2016, economic growth has been higher in the UK than in the Eurozone |
| **Real News** | **Fake News** |
| Crime | Crime |
| Assaults on prison staff in England were up a quarter from around 7,160 to 9,000 in the year up to March 2018 | Knife crime is at record levels in Scotland |
| Four square meters of rainforest are destroyed for each gram of cocaine produced | Nearly 25 per cent of 16 to 24-year-olds had used Class A drugs in the last 12 months (2017 to 2018) |
| People killed by a bicycle account for 0.5% of all road deaths in Britain | Men may be taken to court and face serious criminal charges and sentences if they whistle at a woman and she calls the police, if proposals to count misogyny as a hate crime go ahead |
| **Real News** | **Fake News** |
| Health | Health |
| The average NHS spend per person is £2,200 in England and £2,500 in Scotland | In 2017, 27% of adults exhibited signs of a possible psychiatric disorder |
| Every four minutes a young person (aged 15-24) is diagnosed with gonorrhea or chlamydia | Drug deaths per 1000 of the population are as high for the most deprived cohort as they are for the ever-homeless cohort |
| Childhood diabetes is up 40% in four years | One in four girls or women are in period poverty and are unable to afford sanitary products |
| **Real News** | **Fake News** |
| Immigration | Immigration |
| If you want to marry and bring in someone from a country outside of the EU, you have to have a salary of over £18,600 | Excluding Irish nationals, there are an estimated 13.5 million people living in the UK who are citizens of another EU country |
| 30% of young people in Scotland think immigration should be decreased or stopped completely | There has been a Brexodus of EU citizens since the referendum |
| Illegal immigrants cannot collect benefits | Every 5 minutes, 70 children will be born in the UK, 20 to mothers not born here |
| **Real News** | **Fake News** |
| Education | Education |
| 66% of pupils in England were in good or outstanding schools in 2010 compared to 88% in 2018 | All full-time teachers in Scotland earn over £30,000 per year |
| A higher proportion of applicants from North-East England got into Oxford University between 2015 and 2017 than applicants from London | The University of Strathclyde is a top 10 University for Research Intensity |
| Student loans don’t actually work like loans | Eligible young people in Scotland get £150 per week (Educational Maintenance Allowance) to stay on in post-16 education |
| **Real News** | **Fake News** |
| Scotland | Scotland |
| Spending on public services in Scotland is 20% higher per head than in England | According to the SNPs Sustainable Growth Commission Report an Independent Scotland would have to make sizeable public expenditure reductions |
| As a proportion of GDP, the deficit in Scotland is more than 3 times that of the UK | In 25 years, 60% of the Scottish population will be over 65 |
| According to the SNPs Sustainable Growth Commission Report an Independent Scotland would have to keep the pound for an extended transition period | 47% of full-time research staff at Scottish Universities are non-UK EU nationals |
| **Real News** | **Fake News** |
| Europe | Europe |
| The European Court of Justice (ECJ) powers will continue in the UK during the proposed transition period | Within 18 months of the EU referendum result, 450,000 jobs were lost in the UK |
| The EU referendum was advisory | The Governor of the Bank of England admitted that post-Brexit trade deals could lead to a golden age of trade |
| The final trade deal between the EU and the UK on any future relationship beyond the transition period can be vetoed by any of the 27 EU Member States | If we opt for no deal we will still have a transition period inside the EU until December 2020 |
